# Enlargement of the Colon

**Published:** 1867-08

**Authors:** Wm. Lewitt

**Affiliations:** Demonstrator of Anatomy in Rush Medical College


### ENLARGEMENT OF THE COLON.

By Wm. Lewitt, M.D., Demonstrator of Anatomy in Rush Medical College.

In September, 1864, I was called upon, in company with Dr. E. Wells, of Ann Arbor, Mich., to visit Wm. C., *æt.* 21, to relieve him of what was called constipation. He had not had an evacuation of the bowels for three weeks, during which time he was in the hands of a *spiritual doctor*, who said he was suffering from *windy dropsy*.

On visiting the patient, we found him suffering intense agony from pain in the abdomen, with frequent desire to expel flatus from the rectum, which could only be accomplished by standing upon his head and hands, in a perpendicular position.

On examining the patient, we found the abdomen enormously distended, and so tense that it was impossible to feel the outlines of any of the abdominal organs. We learned from him that he once suffered from a similar attack, when about 12 years of age, which continued for about two weeks, without any evacuation of the bowels, and had ever since then been suffering from torpor of the bowels, only having an evacuation once in eight or ten days.

Upon an examination per anum, we found what appeared to be an enormous tumor, filling up the entire pelvic cavity, and having all the appearance of a child's head at term; in fact, it bore a closer resemblance to that than anything else. The rectum appeared to be normal.

He having taken large quantities of powerful cathartics, it was not deemed advisable to repeat them, and copious enemas, containing ol. terebinth., were ordered, but, in attempting to use them, they could not be thrown up higher than the rectum, and would flow immediately back. It was then thought advisable to try what could be done with the aid of the long rectum tube. This could not be carried farther up than the upper portion of the rectum, when it would fold upon itself, showing that there was some obstruction at the upper end of the rectum or sigmoid flexure of the colon.

Laxatives were used, containing large quantities of fel. bov. and ol. terebinth., but with no effect, with the exception of, occasionally, an expulsion of small quantities of flatus from the rectum, and then only by resorting to the position mentioned above. He continued in this condition for about a week after I saw him, when he was suddenly seized with excruciating pain in the abdomen, and in a few hours expired.

A *post mortem* examination, made about six hours after death, revealed the following condition:—The peritoneal cavity was enormously distended with gas, and a large quantity of fæcal matter, of a battery consistence, was extravasated into it, showing that perforation had taken place, and was the immediate cause of death. Perforation had taken place at several points in the colon. The ascending and descending colon (for there was no transverse) appeared like two immense cylinders, lying side by side, and extending from the epigastrium to the pelvis, and filled with soft fæcal matter, and each was about 5½ inches in diameter. The caput coli was not much enlarged; the transverse colon was entirely obliterated; and the two cylinders of the ascending and descending colon were folded upon themselves, filling up the entire surface of the abdominal cavity, The sigmoid flexure was about the same diameter, and what was supposed to be the tumor filling up the pelvic cavity, was the sigmoid flexure enormously distended with fæcal matter and folded down upon itself, giving it the firm and rounded shape of a pelvic tumor that was supposed to exist, and pressed so firmly upon the upper portion of the rectum as to prevent all passage of fæcal matter into it. The colon was very much thickened and completely filled with fæcal matter of a battery consistence, containing over a large wooden pail full, besides what had extravasated through the perforations into the peritoneal cavity.

The reason for his adopting that peculiar and unnatural position to enable him to expel the flatus from his bowels was, that by that position the weight of the distended sigmoid flexure was taken off the upper portion of the rectum and allowed a small quantity of flatus to escape, which afforded him some relief.

